# Two polymorphisms (rs699947, rs2010963) in the *VEGFA* gene and diabetic retinopathy: an updated meta-analysis

**DOI:** 10.1186/1471-2415-13-56

**Published:** 2013-10-16

**Authors:** Yan Lu, Yirui Ge, Yuhua Shi, Jie Yin, Zhenping Huang

**Affiliations:** 1 Department of Ophthalmology, Jinling Hospital, School of Medicine, Nanjing University, 305 East Zhongshan Road, Nanjing 210002, PR China

**Keywords:** The vascular endothelial growth factor gene, Polymorphism, Diabetic retinopathy, Meta-analysis

## Abstract

**Background:**

The vascular endothelial growth factor (*VEGFA*) gene has been suggested to play an important role in the pathogenesis of diabetic retinopathy (DR). However, the results have been inconsistent. In this study, we performed a meta-analysis to clarify the associations between *VEGFA* polymorphisms and DR risk.

**Methods:**

Published literature from PubMed, EMBASE, Web of Science and Google Scholar were retrieved. Pooled odds ratios (ORs) with 95% confidence intervals (CIs) were calculated using fixed- or random-effects model.

**Results:**

A total of eight studies (1204 cases and 1198 controls) for rs699947 polymorphism and ten studies (1666 cases and 1782 controls) for rs2010963 polymorphism were included in the meta-analysis. The results suggested that rs699947 polymorphism was marginally associated with DR under a homogeneous co-dominant model (AA vs. CC: OR = 1.69, 95% CI = 1.03-2.77, *p* = 0.040) and a dominant model (AA + AC vs. CC: OR = 1.38, 95% CI = 1.01-1.90, *p* = 0.040), whereas the association between rs2010963 polymorphism and DR was not significant under all genetic models (all *p* > 0.05). In the subgroup analysis, the effect size for rs699947 polymorphism was only marginally significant among European populations under a dominant model (OR = 1.47, 95% CI = 1.07–2.02, *p* = 0.018), but not among East Asians. After exclusion of outliers which were the source of between-study heterogeneity, there was significant association between rs699947 polymorphism and DR under a homogeneous co-dominant model (OR = 1.64, 95% CI = 1.18-2.28, *p* = 0.003), even after multiple comparison correction.

**Conclusions:**

Our meta-analysis confirmed the significant association between rs699947 polymorphism and DR after exclusion of outliers, and rs2010963 polymorphism might be not associated with DR.

## Background

Diabetic retinopathy (DR), a micro-vascular complication of diabetes, is a main cause of blindness in adults [1]. It is well established that DR is determined by both genetic and environmental factors. The longer duration of diabetes, poorer control of blood glucose and elevated blood pressure are the major risk factors in the development of DR. However, genetic factors also play important roles in the pathogenesis of DR [2]. It would be useful to identify molecular markers that may help to predict the development of DR at earlier stages of diabetes.

Vascular endothelial growth factor (VEGFA), an endothelial cellspecific mitogen, has been implicated as a major contributor to the development of DR [3]. VEGFA could induce the earliest changes in DR including leukostasis, blood-retinal barrier breakdown, and macular edema and neovascularization in progression of DR [4]. The *VEGFA* gene is located on chromosome 6p21.3 and consists of 8 exons. The genetic variants in the *VEGFA* gene are suggested to influence levels of VEGFA protein expression. To date, many studies have investigated the associations between polymorphisms in the *VEGFA* gene and DR. -634G/C (+405G/C or rs2010963) polymorphism in the 5′-untranslated region and-2578C/A (−2549I/D (in high linkage disequilibrium with -2578C/A) or rs699947) polymorphism in the promoter region of the *VEGFA* gene are most frequently investigated. However, the results have been inconsistent [5-16]. To date, three relevant meta-analyses [17-19] have published. However, only two studies for rs699947 polymorphism and five studies for rs2010963 were included in the meta-analysis by Abhary et al. [17]. Only 6 studies for rs2010963 were included in the meta-analysis by Zhao et al. [18]. Although Qiu et al. [19] found the positive association between rs2010963 polymorphism and DR, it should be noted that the association was only marginally significant as *p* value under allelic model and recessive model was 0.03. Most importantly, the recent meta-analysis by Qiu et al. [19] omitted two studies [7,16] and included one study [20] where the genotype frequency of rs2010963 polymorphism was not in Hardy-Weinberg Equilibrium (HWE) in controls. Thus, the findings from the meta-analysis by Qiu et al. [19] were inaccurate. Most recently, a number of additional new articles have been published. Therefore, in this study, we performed an updated meta-analysis with 8 studies for rs699947 polymorphism and 10 studies for rs2010963 to further clarify the associations.

## Methods

### Literature and search strategy

We searched PubMed, EMBASE, Web of Science and Google Scholar literature databases. The search strategy to identify all possible studies involved the use of the following key words: (the vascular endothelial growth factor OR *VEGFA* OR *VEGF*) and (variant OR variation OR polymorphism OR single nucleotide polymorphism OR SNP) and (diabetic retinopathy OR DR OR PDR). All related studies published in English language were included. The reference lists of retrieved articles were hand-searched. If more than one article were published using the same case series, only the study with the latest data was included. The literature search was updated on July 20, 2013.

### Inclusion criteria and data extraction

The studies included in the meta-analysis met all the following inclusion criteria: (1) evaluated the association of *VEGFA* polymorphism (rs699947 or rs2010963) with DR; (2) used case–control or cohort design; and (3) provided sufficient data for calculation of odds ratio (OR) with 95% confidence interval (CI). The following information was extracted from each study: (1) name of the first author; (2) year of publication; (3) origin of country; (4) sample sizes of cases and controls; (5) genotype distributions of cases and controls; and (6) whether the variant was in HWE in controls. Two authors independently searched articles, assessed the articles for compliance with the inclusion/exclusion criteria, resolved disagreements and reached a consistent decision.

### Statistical analysis

The association of *VEGFA* polymorphism with DR was estimated by calculating pooled ORs and 95% CIs under a co-dominant, a dominant, and a recessive model, respectively. The significance of pooled OR was determined by Z test (*p* < 0.05 was considered statistically significant). Q test was performed to evaluate the between-study heterogeneity. A random- (DerSimonian-Laird method [21] or fixed- (Mantel–Haenszel method) [22] effects model was used to calculate pooled OR in the presence (*p* ≤ 0.10) or absence (*p* > 0.10) of heterogeneity, respectively. Subgroup analyses by ethnicity and type of DR were performed. Sensitivity analysis was performed after excluding one study at a time to test the stability of the result. Begg’s funnel plot, a scatter plot of effect against a measure of study size, was generated as a visual aid to detect bias or systematic heterogeneity [23]. Publication bias was assessed by Egger’s test [24] (*p* < 0.05 was considered statistically significant). Data analysis was performed using STATA version 11 (StataCorp LP, College Station, TX, USA). In this meta-analysis, we used four genetic models for each of two polymorphisms, and Bonferroni method was used to correct for multiple comparisons (*p* = 0.05/8 = 0.00625).

## Results

### Characteristics of the studies

**Figure 1:**
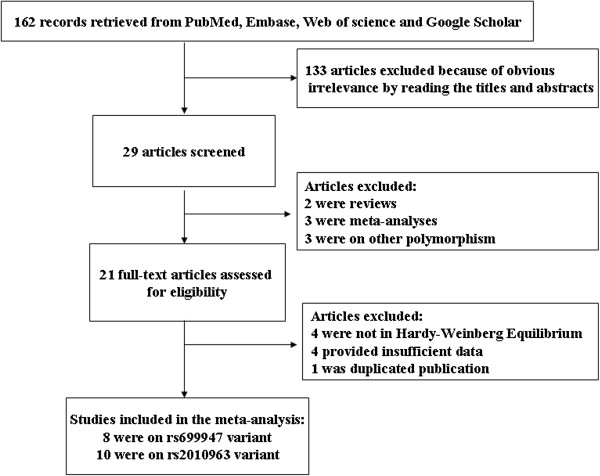
Flow chart of inclusion/exclusion of the individual studies.

The literature search identified a total of 162 potentially relevant papers. Of these, 133 papers were excluded because of obvious irrelevance by reading the titles and abstracts. In addition, two reviews [1,25], three meta-analyses [17-19], and three article [26-28] which investigated the association of other polymorphism in the *VEGFA* gene with DR were excluded. Then, 21 papers met the primary inclusion criteria. However, one duplicated publication [29], four papers [30-33] which provided insufficient data for calculation of OR with 95% CI were further excluded. In addition, because the variant was not in HWE in controls, one article [15] for rs699947 polymorphism and three articles [20,34,35] for rs2010963 polymorphism were excluded. Additionally, since two studies were included in the article by Abhary et al. [8], they were considered as separate studies in the meta-analysis. At last, a total of 8 studies for rs699947 polymorphism and 10 studies for rs2010963 polymorphism were included in the final meta-analysis. A flow chart describing the study inclusion/exclusion is displayed as Figure 1. The characteristics of the included studies are listed in Tables 1 and 2.

**Table 1:** **Characteristic of the studies included in the meta-analysis between rs699947 polymorphism in the*VEGFA*gene and diabetic retinopathy**

**Study**	**Country**	**Ethnicity**	**Sample size**		**Genotype frequency in cases**			**Genotype frequency in controls**			***P***_**HWE**_
			**Cases**	**Controls**	**CC**	**AC**	**AA**	**CC**	**AC**	**AA**	
Yang,2003 [5]	UK	European	64	66	12	31	21	13	38	15	Yes
Awata,2005 [6]	Japan	East Asian	175	203	95	70	10	93	91	19	Yes
Buraczynska,2007 [7]	Poland	European	195	91	29	80	86	29	43	19	Yes
Abhary,2009 [8]	Australia	European	75	93	17	35	23	26	43	24	Yes
Abhary,2009 [8]	Australia	European	136	181	31	74	31	45	91	45	Yes
Nakamura,2009 [9]	Japan	East Asian	177	292	85	70	22	163	107	22	Yes
Chun,2010 [10]	Korea	East Asian	253	134	123	115	15	92	36	6	Yes
Yang,2011 [11]	China	East Asian	129	138	66	47	16	82	51	5	Yes

**Table 2:** **Characteristic of the studies included in the meta-analysis between rs2010963 polymorphism in the*VEGFA*gene and diabetic retinopathy**

**Study**	**Country**	**Ethnicity**	**Sample size**		**Genotype frequency in cases**			**Genotype frequency in controls**			***P***_**HWE**_
			**Cases**	**Controls**	**GG**	**GC**	**CC**	**GG**	**GC**	**CC**	
Awata,2005 [6]	Japan	East Asian	175	200	46	91	38	75	95	30	Yes
Buraczynska,2007 [7]	Poland	European	195	91	88	88	19	42	41	8	Yes
Errera,2007 [12]	Brazil	European	167	334	57	73	37	139	155	40	Yes
Petrovic,2008 [13]	Slovenia	European	206	143	79	103	24	61	67	15	Yes
Uthra,2008 [14]	India	South Asian	120	79	60	51	9	44	29	6	Yes
Nakamura,2009 [9]	Japan	East Asian	176	289	63	79	34	84	146	59	Yes
Kangas-Kontio,2009 [15]	Finland	European	126	96	74	42	10	52	39	5	Yes
Chun,2010 [10]	Korea	East Asian	253	134	85	125	43	43	69	22	Yes
Feghhi,2011 [16]	Iran	European	119	279	43	49	27	63	139	77	Yes
Yang,2011 [11]	China	East Asian	129	137	36	74	19	39	72	26	Yes

### Meta-analysis results

**Figure 2:**
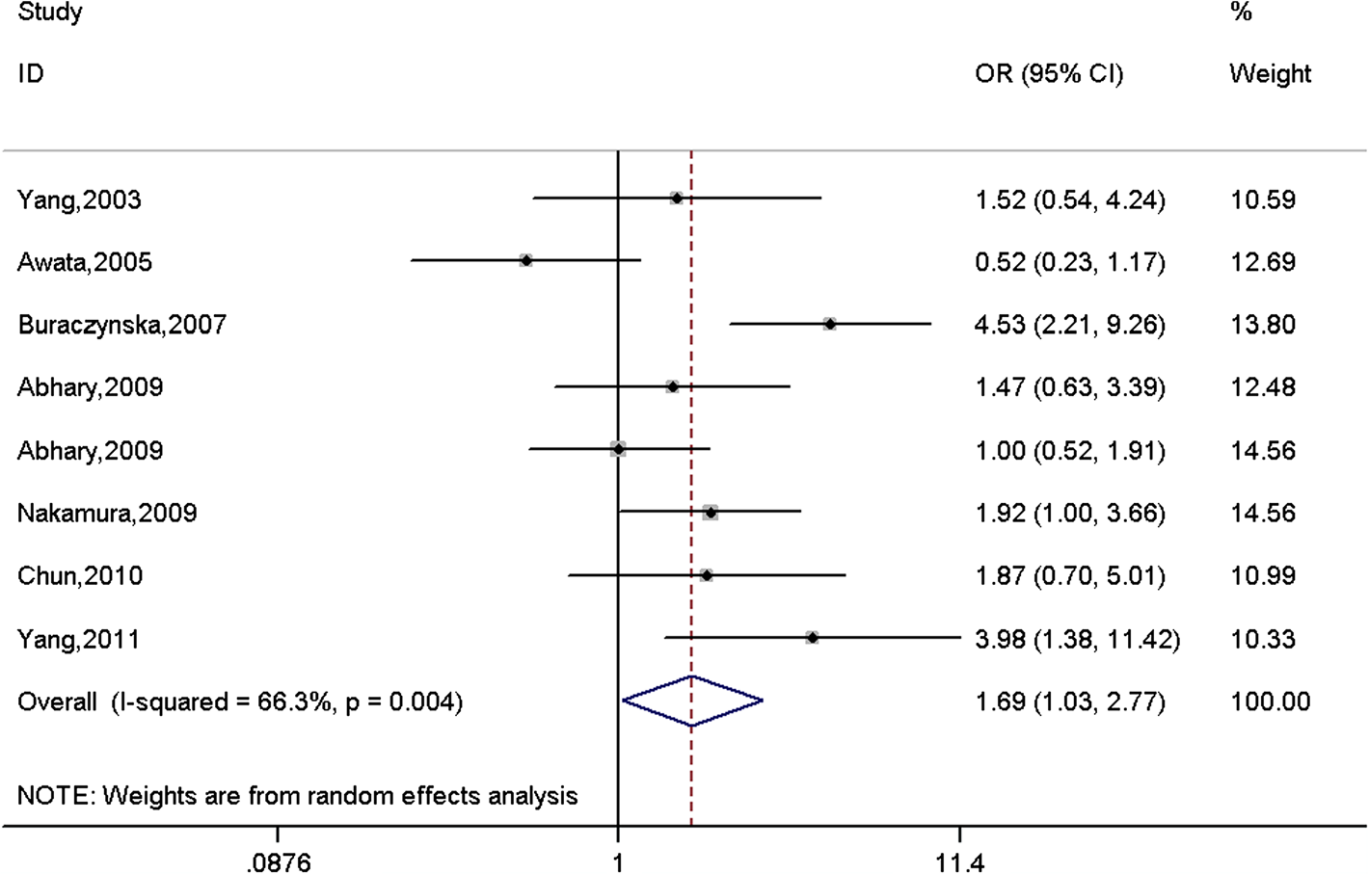
**Meta-analysis of the association between rs699947 polymorphism in the *VEGFA *gene and diabetic retinopathy under a homogeneous co-dominant model (AA vs. CC).**

A total of 1204 cases and 1198 controls were identified for the association between rs699947 polymorphism and DR. The results indicated that rs699947 polymorphism was marginally associated with DR under a homogeneous co-dominant model (AA vs. CC: OR = 1.69, 95% CI = 1.03-2.77, *p* = 0.040, Figure 2) and a dominant model (AA + AC vs. CC: OR = 1.38, 95% CI = 1.01-1.90, *p* = 0.040). Further subgroup analysis suggested that the effect size was only significant among European populations under a dominant model (AA + AC vs. CC: OR = 1.47, 95% CI = 1.07–2.02, *p* = 0.018), but not among East Asian populations under all genetic models (Table 3). Since only two studies for proliferative DR (PDR) and one study for nonproliferative DR (NPDR) were investigated, the subgroup analysis by type of DR were not performed.

**Table 3:** **Pooled ORs and 95% CIs of the association between rs699947 polymorphism in the*VEGFA*gene and diabetic retinopathy**

**Contrasts**	**No. of studies (cases/controls)**	**Homogeneous co-dominant model**				**Heterogeneous co-dominant model**				**Dominant model**				**Recessive model**			
		**AA vs. CC**				**AC vs. CC**				**AA + AC vs. CC**				**AA vs. AC + CC**			
		**OR**	**95% CI**	***I***^**2**^**(%)**	***P***_**H**_	**OR**	**95% CI**	***I***^**2**^**(%)**	***P***_**H**_	**OR**	**95% CI**	***I***^**2**^**(%)**	***P***_**H**_	**OR**	**95% CI**	***I***^**2**^**(%)**	***P***_**H**_
All	8 (1204/1198)	1.69	1.03-2.77 ^R^	66.3	0.004	1.27	0.96-1.69 ^R^	54.2	0.032	1.38	1.01-1.90 ^R^	67.1	0.003	1.49	0.99-2.24 ^R^	61.5	0.011
Ethnicity																	
European	4 (470/431)	1.79	0.87-3.67 ^R^	69.7	0.020	1.31	0.93-1.83 ^F^	0.0	0.565	1.47	1.07-2.02 ^F^	46.4	0.133	1.53	0.87-2.68 ^R^	68.6	0.023
East Asian	4 (734/767)	1.59	0.71-3.57 ^R^	71.7	0.014	1.26	0.79-2.02 ^R^	77.3	0.004	1.33	0.82-2.14 ^R^	80.3	0.002	1.46	0.72-2.96 ^R^	65.1	0.035

R, random-effect model; F, fixed-effect model.

**Figure 3:**
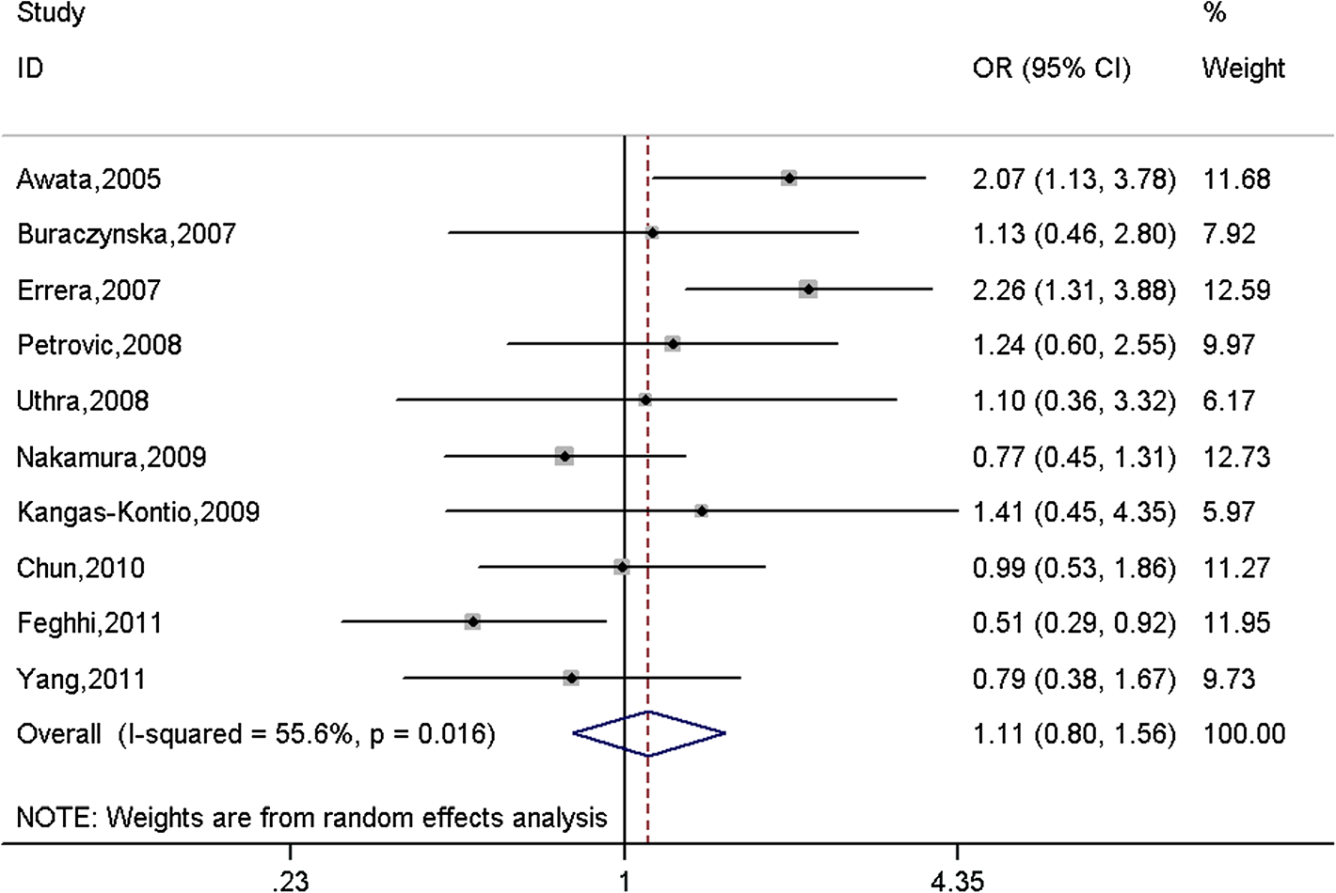
**Meta-analysis of the association between rs2010963 polymorphism in the *VEGFA *gene and diabetic retinopathy under a homogeneous co-dominant model (CC vs. GG).**

A total of 1666 cases and 1782 controls were identified for the association between rs2010963 polymorphism and DR. The results showed that there was no significant association between this polymorphism and DR under all genetic models (Table 4 and Figure 3), even after stratified for ethnicity and type of DR.

**Table 4:** **Pooled ORs and 95% CIs of the association between rs2010963 polymorphism in the*VEGFA*gene and diabetic retinopathy**

**Contrasts**	**No. of studies (cases/controls)**	**Homogeneous co-dominant model**				**Heterogeneous co-dominant model**				**Dominant model**				**Recessive model**			
		**CC vs. GG**				**CG vs. GG**				**CC + CG vs. GG**				**CC vs. CG + GG**			
		**OR**	**95% CI**	***I***^**2**^**(%)**	***P***_**H**_	**OR**	**95% CI**	***I***^**2**^**(%)**	***P***_**H**_	**OR**	**95% CI**	***I***^**2**^**(%)**	***P***_**H**_	**OR**	**95% CI**	***I***^**2**^**(%)**	***P***_**H**_
All	10 (1666/1782)	1.11	0.80-1.56 ^R^	55.6	0.016	0.98	0.80-1.20 ^R^	40.6	0.087	1.01	0.81-1.26 ^R^	54.4	0.020	1.13	0.93-1.37 ^F^	28.1	0.186
Ethnicity																	
European	5 (813/943)	1.17	0.64-2.13 ^R^	70.0	0.010	0.90	0.66-1.23 ^R^	49.5	0.094	0.95	0.67-1.35 ^R^	65.0	0.022	1.25	0.81-1.92 ^R^	50.6	0.088
East Asian	4 (733/760)	1.06	0.67-1.69 ^R^	55.4	0.081	1.02	0.73-1.44 ^F^	50.3	0.110	1.04	0.72-1.49 ^R^	60.9	0.053	1.05	0.80-1.38 ^F^	17.1	0.306
South Asian	1 (120/79)	1.10	0.36-3.32	-	-	1.29	0.71-2.35	-	-	1.26	0.71-2.22	-	-	0.99	0.34-2.89	-	-
Type of DR																	
PDR	7 (924/1376)	1.14	0.71-1.83 ^R^	68.0	0.005	0.91	0.75-1.10 ^F^	34.2	0.167	0.97	0.73-1.28 ^R^	56.9	0.030	1.22	0.86-1.74 ^R^	53.5	0.045
NPDR	3 (267/331)	1.74	0.70-4.29 ^R^	62.9	0.067	1.45	0.73-2.87 ^R^	70.5	0.034	1.50	0.74-3.03 ^R^	74.5	0.020	1.36	0.85-2.18 ^F^	0.0	0.492

R, random-effect model; F, fixed-effect model.

### Source of heterogeneity

**Figure 4:**
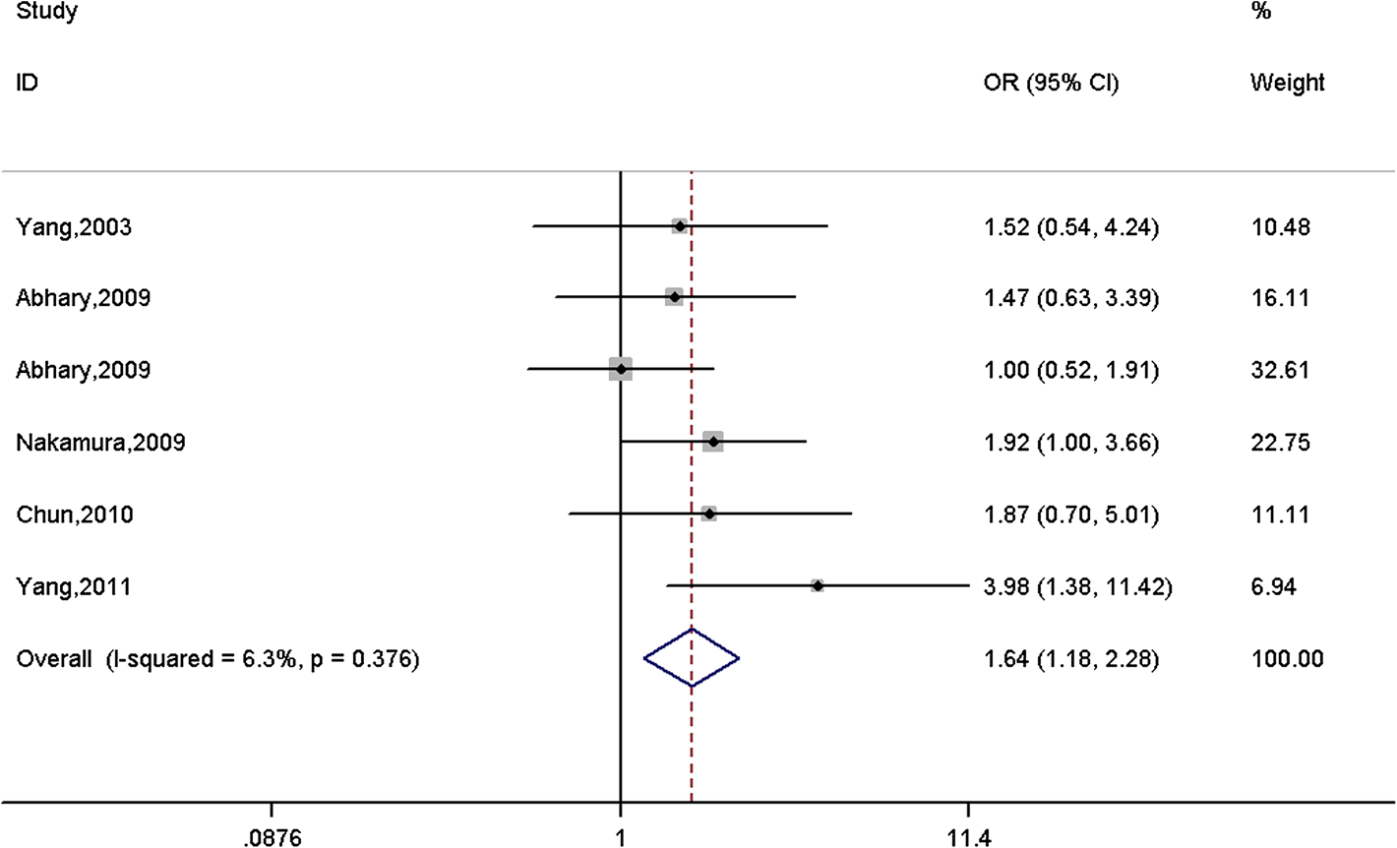
**Meta-analysis of the association between rs699947 polymorphism in the *VEGFA *gene and diabetic retinopathy after exclusion of outliers under a homogeneous co-dominant model (AA vs. CC).**

Since there was significant between-study heterogeneity for both rs699947 and rs2010963 polymorphisms, we performed a meta-regression analysis to explore source of heterogeneity. We introduced variables including publication year, ethnicity, sample size in cases and controls. However, these variables can not explain the source of heterogeneity. Then, we drew Galbraith figure to further explore the outliers. The pooled results after exclusion of these outliers are listed in Additional file 1: Table S1. The between-study heterogeneity for two polymorphisms disappeared, and non-significant association for rs2010963 polymorphism with DR remained (Additional file 1: Table S1). However, the association between rs699947 polymorphism and DR changed to be significant under homogeneous co-dominant model (OR = 1.64, 95% CI = 1.18-2.28, *p* = 0.003, Figure 4), even after sensitivity analysis was performed and multiple comparison correction was applied.

### Potential publication bias

Using the Egger’s test, no publication bias was detected for the studies on rs699947 polymorphism and rs2010963 polymorphism under all genetic models (all *p* > 0.05).

## Discussion

The present updated meta-analysis confirmed the significant association between rs699947 polymorphism and DR after exclusion of outliers, and rs2010963 polymorphism might be not associated with DR. In the subgroup analysis, although rs699947 polymorphism was marginally associated with DR among Europeans under a dominant model, it did not reach statistical significance after multiple comparison correction.

To date, a number of studies have investigated association between VEGFA gene variants and risk of DR. Awata et al. [6] first reported that rs2010963 polymorphism was significantly associated with DR in Japanese patients with type 2 diabetes. However, the following studies demonstrated inconsistent results. Another polymorphisms (i.e. rs699947) has also been investigated and the conflicting results were also reported. The discrepant findings might be due to differences in the statistical power, the recruitment of studied population, the type of DR, and the genetic and environmental backgrounds.

In the present meta-analysis, there was evidence of between-study heterogeneity for both rs699947 and rs2010963 polymorphisms. The meta-regression analysis was performed to explore source of heterogeneity. However, variables such as publication year, ethnicity, sample size in cases and controls can not account for the source heterogeneity. After exclusion of outliers, the heterogeneity for both rs699947 and rs2010963 polymorphisms was abolished. Interestingly, there was significant association between rs699947 polymorphism and DR under a homogeneous co-dominant model (OR = 1.64, 95% CI = 1.18-2.28, *p* = 0.003), even after multiple comparison correction was applied.

The mechanism between *VEGFA* gene and DR is currently unclear. DR is characterized by increased vascular permeability, tissue ischemia and neovascularization. VEGFA can stimulate angiogenesis and increases the permeability of the microvasculature [17]. It has been suggested that VEGFA protein expression is affected by genetic variant in the *VEGFA* gene. Compared with VEGFA levels in the vitreous of diabetic eyes without PDR, those in the vitreous of patients with PDR are significantly elevated [36]. In addition, VEGFA inhibition has been indicated to cause a marked reduction in retinal neovascularization and prevention of the blood–retinal barrier breakdown [4].

Several limitations should be noted. First, the present meta-analysis was based primarily on unadjusted ORs with 95% CIs and the confounding factors (e.g. age, sex, duration of diabetes and hypertension) were not controlled for. Second, the effects of gene–gene and gene–environment interactions were not addressed in this meta-analysis since the original publications did not provide the related data. Third, the results in the subgroups should be interpreted with caution because of limited sample size. Fourth, for rs699947 polymorphism, since majority of included studies did not report the association with type of DR, we are unable to perform the further analysis. Fifth, we only assessed two polymorphisms in the *VEGFA* gene, therefore, we can not rule out the possibility that other polymorphisms or haplotypes in this gene might be implicated in the development of DR.

## Conclusions

In conclusions, the results of our meta-analysis indicated that there was a significantly association between *VEGFA* rs699947 polymorphism and risk of DR after exclusion of outliers, whereas rs2010963 polymorphism might be not associated with risk of DR. Further well-designed large-scale studies with the consideration of gene–gene and gene–environment interactions should be conducted to investigate the association in future.

## Competing interests

The authors declare that they have no competing interests.

## Authors’ contributions

Conceived and designed the study: ZH. Acquisition of data: YS JY. Analysis and interpretation of data: YL, YG, ZH. Drafting the manuscript: YL, YG. Revising the manuscript critically for important intellectual content: YL, YG, YS, JY, ZH. All authors read and approved the final manuscript.

## Pre-publication history

The pre-publication history for this paper can be accessed here:

http://www.biomedcentral.com/1471-2415/13/56/prepub

## Supplementary Material

Additional file 1: Table S1 Pooled ORs and 95% CIs of the association between *VEGFA* variants and diabetic retinopathy after exclusion of the outliers. Click here for file
