# Supplementary material for: Two polymorphisms (rs699947, rs2010963) in the VEGFA gene and diabetic retinopathy: an updated meta-analysis
Source: BMC Ophthalmol. 2013 Oct 16;13:56. doi: 10.1186/1471-2415-13-56 (PMC3852979; doi:10.1186/1471-2415-13-56)
Supplement: Additional file 1: Table S1 — Pooled ORs and 95% CIs of the association between VEGFA variants and diabetic retinopathy after exclusion of the outliers. [file 1471-2415-13-56-S1.doc]

Table S1 Pooled ORs and 95%CIs of the association between *VEGFA* variants and diabetic retinopathy after exclusion of the outliers

| Variant | Homogeneous co-dominant model | | | | | Heterogeneous co-dominant model | | | | | Dominant model | | | | | Recessive model | | | | |
| --- | --- | --- | --- | --- | --- | --- | --- | --- | --- | --- | --- | --- | --- | --- | --- | --- | --- | --- | --- | --- |
|  | Excluded studies | OR | 95%CI | *I*^2^  (%) | *P* _H_ | Excluded studies | OR | 95%CI | *I*^2^  (%) | *P* _H_ | Excluded studies | OR | 95%CI | *I*^2^  (%) | *P* _H_ | Excluded studies | OR | 95%CI | *I*^2^  (%) | *P* _H_ |
| rs699947 | [6,7] | 1.64 | 1.18-2.28 | 6.3 | 0.38 | [6, 10] | 1.25 | 1.00-1.58 | 0.0 | 0.82 | [6,7,10] | 1.29 | 1.02-1.63 | 0.0 | 0.95 | [6,7] | 1.40 | 1.06-1.86 | 29.6 | 0.21 |
| rs2010963 | [12,16] | 1.10 | 0.86-1.42 | 0.9 | 0.42 | [16] | 1.05 | 0.89-1.23 | 4.2 | 0.40 | [6,16] | 1.03 | 0.88-1.21 | 0.0 | 0.469 | [16] | 1.02 | 0.83-1.25 | 0.0 | 0.69 |
